# Variation potential influence on photosynthetic cyclic electron flow in pea

**DOI:** 10.3389/fpls.2014.00766

**Published:** 2015-01-07

**Authors:** Vladimir Sukhov, Lyubov Surova, Oksana Sherstneva, Lyubov Katicheva, Vladimir Vodeneev

**Affiliations:** Department of Biophysics, N.I. Lobachevsky State University of Nizhny NovgorodNizhny Novgorod, Russia

**Keywords:** cyclic electron flow, noncyclic electron flow, *Pisum sativum*, photosynthetic dark stage, photosystem I, photosystem II, variation potential

## Abstract

Cyclic electron flow is an important component of the total photosynthetic electron flow and participates in adaptation to the action of stressors. Local leaf stimulation induces electrical signals, including variation potential (VP), which inactivate photosynthesis; however, their influence on cyclic electron flow has not been investigated. The aim of this study was to investigate VP's influence on cyclic electron flow in pea (*Pisum sativum* L.). VP was induced in pea seedling leaves by local heating and measured in an adjacent, undamaged leaf by extracellular electrodes. CO_2_ assimilation was measured using a portable gas exchange measuring system. Photosystem I and II parameters were investigated using a measuring system for simultaneous assessment of P700 oxidation and chlorophyll fluorescence. Heating-induced VP reduced CO_2_ assimilation and electron flow through photosystem II. In response, cyclic electron flow rapidly decreased and subsequently slowly increased. Slow increases in cyclic flow were caused by decreased electron flow through photosystem II, which was mainly connected with VP-induced photosynthetic dark stage inactivation. However, direct influence by VP on photosystem I also participated in activation of cyclic electron flow. Thus, VP, induced by local leaf-heating, activated cyclic electron flow in undamaged leaves. This response was similar to photosynthetic changes observed under the direct action of stressors. Possible mechanisms of VP's influence on cyclic flow were discussed.

## Introduction

Photosynthesis in plants is based on three light-driven electron flows, namely noncyclic, pseudocyclic, and cyclic flows (Allen, 2003). Cyclic electron flow is connected with photosystem I (PSI) and cytochrome b_6_f (Joliot and Joliot, 2006; Joliot and Johnson, 2011; Roach and Krieger-Liszkay, 2014), whereas other flows are also connected with photosystem II (PSII) (Allen, 2003; Roach and Krieger-Liszkay, 2014). In contrast to noncyclic and pseudocyclic electron flows, cyclic flow only yields ATP synthesis and does not generate NADPH for the Calvin cycle or reactive oxygen species (Allen, 2003).

Cyclic electron flow can be activated by different stressors and might serve as an adaptive mechanism (Bukhov et al., 1999; Rumeau et al., 2007; Zhang and Sharkey, 2009; Zivcak et al., 2013). In particular, under stress conditions, cyclic flow might regulate photosynthetic generation of reactive oxygen species (Zhang and Sharkey, 2009; Roach and Krieger-Liszkay, 2014), contribute to oxidation of the PSI acceptor side, thereby protecting it from damage (Rumeau et al., 2007; Roach and Krieger-Liszkay, 2014), and support the transthylakoid proton gradient (Bukhov et al., 1999; Joliot and Joliot, 2006; Zhang and Sharkey, 2009). In turn, support of the pH gradient contributes to ATP synthesis, fluorescence non-photochemical quenching (NPQ), and thylakoid membrane stability (Zhang and Sharkey, 2009; Joliot and Johnson, 2011). Thus, it can be concluded (Roach and Krieger-Liszkay, 2014) that activation of cyclic electron flow by the direct action of stressors plays an important role in adaptive responses in plants.

Local action by stressors induces electrical signals, namely action potential (AP) induced by non-damaging stimuli and variation potential (VP) caused by damaging stimuli, which propagate through the unstimulated parts of higher plants (Volkov, 2000; Dziubinska, 2003; Brenner et al., 2006; Mancuso and Mugnai, 2006; Stahlberg et al., 2006; Trebacz et al., 2006). Self-propagating AP is mainly caused by fluxes of Ca^2+^, K^+^, and Cl^−^ ions (Felle and Zimmermann, 2007), while transient H^+^-ATPase inactivation and proton influx participate in its generation to a lesser degree (Sukhov and Vodeneev, 2009). VP generation is connected with transient plasmalemma H^+^-ATPase inactivation (Stahlberg et al., 2006); however, fluxes of Ca^2+^, K^+^, and Cl^−^ ions might also participate in VP development (Vodeneev et al., 2011; Sukhov et al., 2013b; Katicheva et al., 2014). VP propagation is probably connected with transmission of hydraulic and/or chemical signals (Stahlberg et al., 2006; Trebacz et al., 2006; Vodeneev et al., 2012), which induce an electrical reaction.

Electrical signals can induce numerous functional responses (Dziubinska, 2003; Davies and Stankovic, 2006; Stahlberg et al., 2006; Volkov et al., 2008; Fromm and Lautner, 2012). In particular, they inactivate photosynthesis in unstimulated leaves (Koziolek et al., 2004; Krupenina and Bulychev, 2007; Grams et al., 2009; Pavlovič et al., 2011; Sukhov et al., 2012, 2013a, 2014a,b; Bulychev and Komarova, 2014). The first stage of photosynthetic response development is possibly ion influx. Investigations of Bulychev and coworkers (Krupenina and Bulychev, 2007) have shown that Ca^2+^ influx is a potential mechanism for AP influence on photosynthesis in *Chara* alga. In regard to VP, according to studies by Grams et al. (2009) and our previous investigations (Sukhov et al., 2013a, 2014a), plasmalemma H^+^-ATPase inactivation and subsequent proton influx are main mechanisms for VP influence on photosynthesis in higher plants.

Independent of mechanisms for initial photosynthetic-response induction, subsequent development of this response is mainly connected with inactivation of the photosynthetic dark stage (Krupenina and Bulychev, 2007; Pavlovič et al., 2011; Pavlovič, 2012; Sukhov et al., 2012, 2014a,b), which decreases quantum yields of photosystem I and II and increases non-photochemical fluorescence quenching. Influence of Ca^2+^ influx on photosynthetic dark stage can be connected (Krupenina and Bulychev, 2007) with the dependence of Calvin cycle enzymes on calcium concentrations in chloroplast stroma (Wolosiuk et al., 1993). Proton influx into cytoplasm and stroma can influence CO_2_ transport, changing carbonic anhydrase (Grams et al., 2009) and/or aquaporin (Gallé et al., 2013) activities and modifying the CO_2_/HCO^−^_3_ ratio (Bulychev et al., 2001). This influx might also reduce Calvin cycle activity (Wolosiuk et al., 1993). However, the direct influence of AP and VP on the light stage is also possible (Pavlovič et al., 2011; Sukhov et al., 2012, 2014a,b). The influence can be related to the rise of fluorescence non-photochemical quenching (Sukhov et al., 2014a) and reduced electron flow through the acceptor side of PSI (Sukhov et al., 2012), which might be caused by acidification of the stroma and lumen (Müller et al., 2001; Alte et al., 2010; Benz et al., 2010).

According to Retivin et al. (1997), rapid and transient increases in plant resistance to stressors (10–25 min after stimulation) are the final result of electrical signal-induced functional responses. The resistance increase in unstimulated parts of plant contributes to plant survival under systemic action of stressor which may follow after electrical signal induction (Retivin et al., 1997). Decrease of photosynthetic machinery damage can be a mechanism contributing to the influence of electrical signals on plant resistance to stressors (Sukhov et al., 2014b). AP (Retivin et al., 1999) and VP (Sukhov et al., 2014b) increase the resistance of photosynthetic machinery to the effects of temperature changes. Our previous results (Sukhov et al., 2014b) have shown that increased resistance of photosynthetic machinery to heat is caused by VP-induced inactivation of the photosynthetic dark stage. Changes in cyclic electron flow can link the VP-induced dark stage inactivation and photosynthetic machinery resistance increase (Sukhov et al., 2014b). However, experimental investigations of electrical signal influence on cyclic electron flow are lacking. Thus, the aim of the present study was to investigate VP influence on cyclic electron flow in pea (*Pisum sativum* L.).

## Materials and methods

### Plant material

Seedlings of pea (*Pisum sativum* L.) were cultivated hydroponically in a Binder KBW 240-plant growth chamber (Binder GmbH, Tuttlingen, Germany) at 24°C under a 16/8 h (light/dark) photoperiod. Seedlings used in experiments were 14–21 days old.

### Stimulation and electrical measurements

VP was induced by heating ~1 cm^2^ of a leaf tip (a stimulated leaf) over a flame for 3–4 s, representing a standard damaging stimulus (Koziolek et al., 2004; Vodeneev et al., 2012; Sukhov et al., 2014a,b).

**Figure 1:**
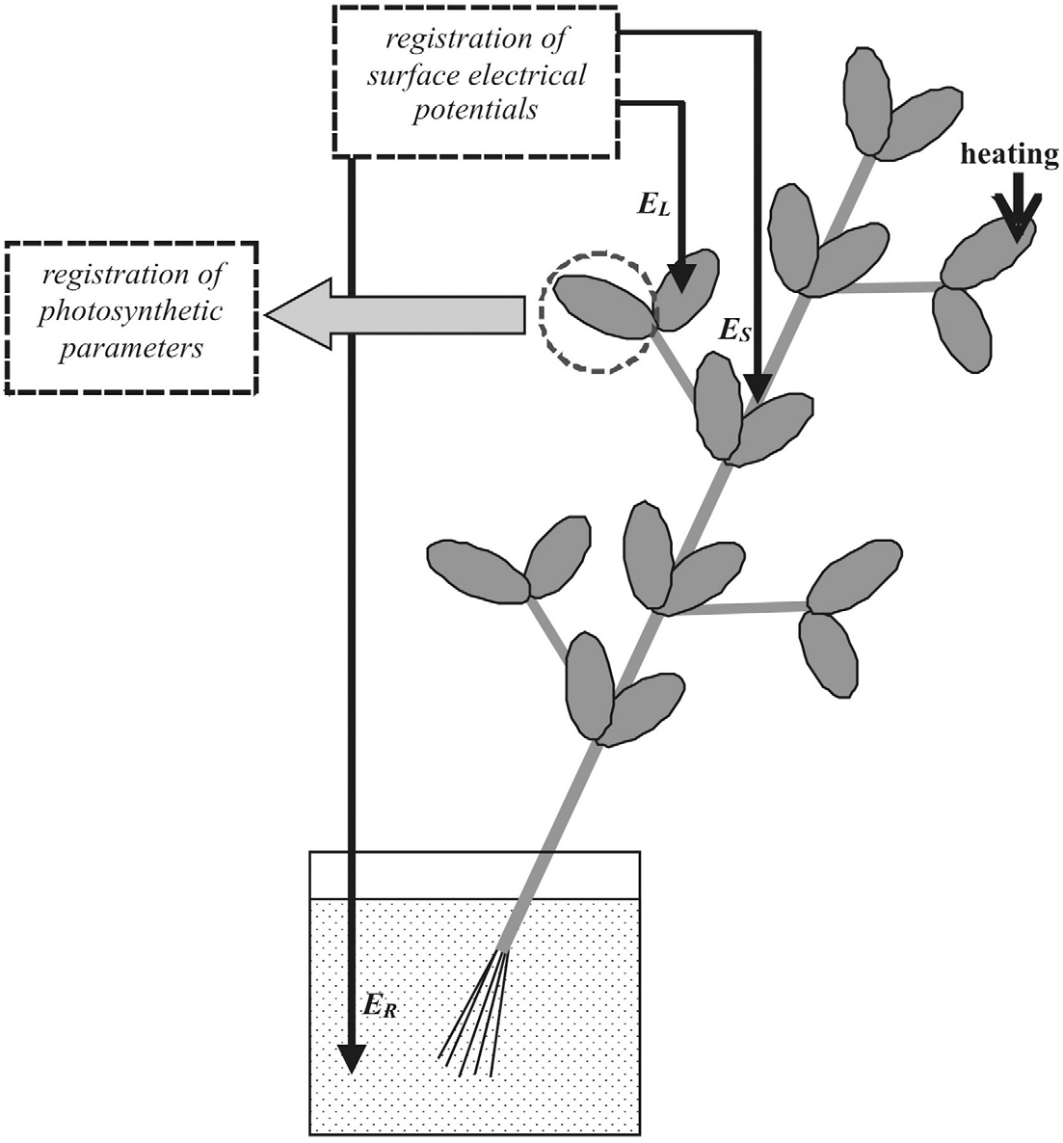
**Positions of stimulation, electrical potential, and photosynthetic parameter measurements in pea plants**. *E*_*L*_, electrode connected to leaf; *E*_*S*_, electrode connected to stem; and *E*_*R*_, reference electrode; distance between *E*_*L*_ and *E*_*S*_, 3–5 cm; and heating of leaf tip (arrow) used as external stimulus.

The surface electrical potential was measured using Ag^+^/AgCl electrodes (Gomel Plant of Measuring Equipment, Gomel, Belarus), a high-impedance amplifier IPL-113 (Semico, Novosibirsk, Russia) and a PC. Measurement electrodes contacted an unstimulated leaf via “Uniagel” conductive gel (Geltek-Medica, Moscow, Russia), according to our previous studies (Sukhov et al., 2014a,b). Electrical activity was monitored by two electrodes (Figure 1), with the first (*E*_*S*_) placed on a stem and the second (*E*_*L*_) connected with a leaflet center of an unstimulated leaf. The distance between the *E*_*S*_ site and the damaged area was 6–7 cm and the distance between *E*_*S*_ and *E*_*L*_ 3–5 cm. It should be noted that, as electrical responses in conjugate leaflets of a leaf were very similar in pea (Sukhov et al., 2014a,b), the electrical reaction, registered by *E*_*L*_, was used for investigation of VP parameters in conjugate leaflets in which photosynthesis was measured. The *E*_*R*_ was placed in a standard solution surrounding the root.

### Measurements of photosynthetic parameters

Photosynthetic parameters in intact pea leaves were measured by a system composed of a GFS-3000 portable gas exchange measuring system, a Dual-PAM-100 measuring system for simultaneous assessment of P700 oxidation and chlorophyll fluorescence, and a measuring head Dual-PAM gas exchange Cuvette 3010-Dual (Heinz Walz GmbH, Effeltrich, Germany). The system was employed for simultaneous recording of photosynthetic dark and light stage parameters in unstimulated leaf lamina (measured area, 1.3 cm^2^).

The initial parameters of PSII fluorescence, the dark and maximal fluorescence yields (*F*_0_ and *F*_*m*_, respectively), were measured after dark adaptation for 20 min. The maximal change in the P700 signal (*P*_*m*_) of PSI, reflecting maximal P700 oxidation, was measured after preliminary illumination by far red light for 10 s. The steady-state fluorescence yields in light (*F* and *F*′_*m*_, respectively), and steady-state and maximal signals in light (*P* and *P*′_*m*_, respectively) were measured using saturation pulses generated every 10 s. Quantum yields of PSI (ϕ_*PSI*_), non-photochemical energy dissipation in PSI because of donor side limitation (ϕ_*ND*_), and non-photochemical energy dissipation in PSI connected with acceptor-side limitation (ϕ_*NA*_) were calculated using the equations ϕ_*PSI*_ = (*P*′_*m*_ − *P*)/*P*_*m*_, ϕ_*ND*_ = *P*/*P*_*m*_, and ϕ_*NA*_ = (*P*_*m*_ − *P*′_*m*_)/*P*_*m*_ (Klughammer and Schreiber, 2008). The effective quantum yield of PSII (ϕ_*PSII*_) and fluorescence non-photochemical quenching (*NPQ*) were calculated using the equations ϕ_*PSII*_ = (*F*′_*m*_ − *F*) / *F*′_*m*_ and *NPQ* = (*F*_*m*_ − *F*′_*m*_) / *F*′_*m*_ (Maxwell and Johnson, 2000). The CO_2_ assimilation rate (*A*, μmol CO_2_·*m*^−2^·*s*^−1^) was measured using the GFS-3000 system and its software, and the parameter programmatically calculated according to Von Caemmerer and Farquhar (1981).

The external CO_2_ concentration ([CO_2_]) was 360 ppm in the control and ~10–15 ppm under low [CO_2_] conditions. In some series of experiments, CO_2_ concentration was decreased from 360 ppm to ~150 or ~10–15 ppm. Relative air humidity and leaf temperature were ~60% and ~23°C, respectively. Blue actinic light (460 nm) intensity in the control was 239 μmol·m^−2^·*s*^−1^. In a separate experimental series, far red light (240 μmol·m^−2^·*s*^−1^, 730 nm) was used as actinic light.

VP was induced in plants ~1 h after initiation of actinic light, and photosynthetic responses monitored for 30 min.

### Calculations of electron flows

Electron flows through PSI [*EF(PSI)*] and PSII [*EF(PSII)*] were calculated using Equations (1) and (2) (Miyake et al., 2004, 2005; Huang et al., 2012; Zivcak et al., 2013):

(1) $$EF(PSI)=\alpha_{I}\times \phi_{PSI}\times PFD,$$

(2) $$EF(PSII)=\alpha_{II}\times \phi_{PSII}\times PFD,$$

where *PFD* was the photosynthetically-active photon flux density of light illuminating a leaf, α_*I*_ = *p* × (1 − *dII*) and α_*II*_ = *p* × *dII* the fractions of photon flux distributed to PSI and PSII, *dII* the fraction of absorbed light distributed to PSII, and *p* the fraction of PFD absorbed by leaves.

The electron flow through PSI included noncyclic, pseudocyclic, and cyclic flows, whereas the electron flow through PSII included only noncyclic and pseudocyclic flows (Allen, 2003). Thus, cyclic electron flow [*EF(C)*] is described as Equation (3) (Miyake et al., 2004, 2005; Huang et al., 2012; Zivcak et al., 2013):

(3) EF(C)=EF(PSI)−EF(PSII).

Calculation of *EF(C)* required values for *p* and *dII* [Equations (1)–(3)]. The value of *p* was measured according to Berger et al. (2004), using a standard procedure in IMAGING-PAM M-Series MINI Version (Heinz Walz GmbH) and found to be 0.88 ± 0.01 (*n* = 10).

According to a number of studies (Miyake and Yokota, 2000; Makino et al., 2002; Miyake et al., 2004, 2005), the fraction of absorbed light distributed to PSII was calculated on the basis of the Farquhar, Von Caemmerer and Berry photosynthetic model of Von Caemmerer et al. (2009). According to this model, CO_2_ assimilation (*A*) under electron transport limited conditions is described by the Equation (4):

(4) $$A=\frac{\left(C_{c}-\Gamma^{*}\right)}{4\left(C_{c}+2\Gamma^{*}\right)}\times EF(PSII)-R_{d},$$

where Γ^*^ is the photosynthetic CO_2_ compensation point in the absence of mitochondrial respiration (36.9–39.6 ppm), *R*_*d*_ the respiration rate in darkness, and *C*_*c*_ the mole fraction of CO_2_ in chloroplasts. Under high [CO_2_] (C_*c*_ → ∞) Equation (4) transforms to Equation (5):

(5) $$EF(PSII)=4\left(A+R_{d}\right),$$

Combining Equations (1) and (3) yields:

(6) $$dII=\frac{4\left(A+R_{d}\right)}{p\times \phi_{PSII}\times PFD},$$

**Figure 2:**
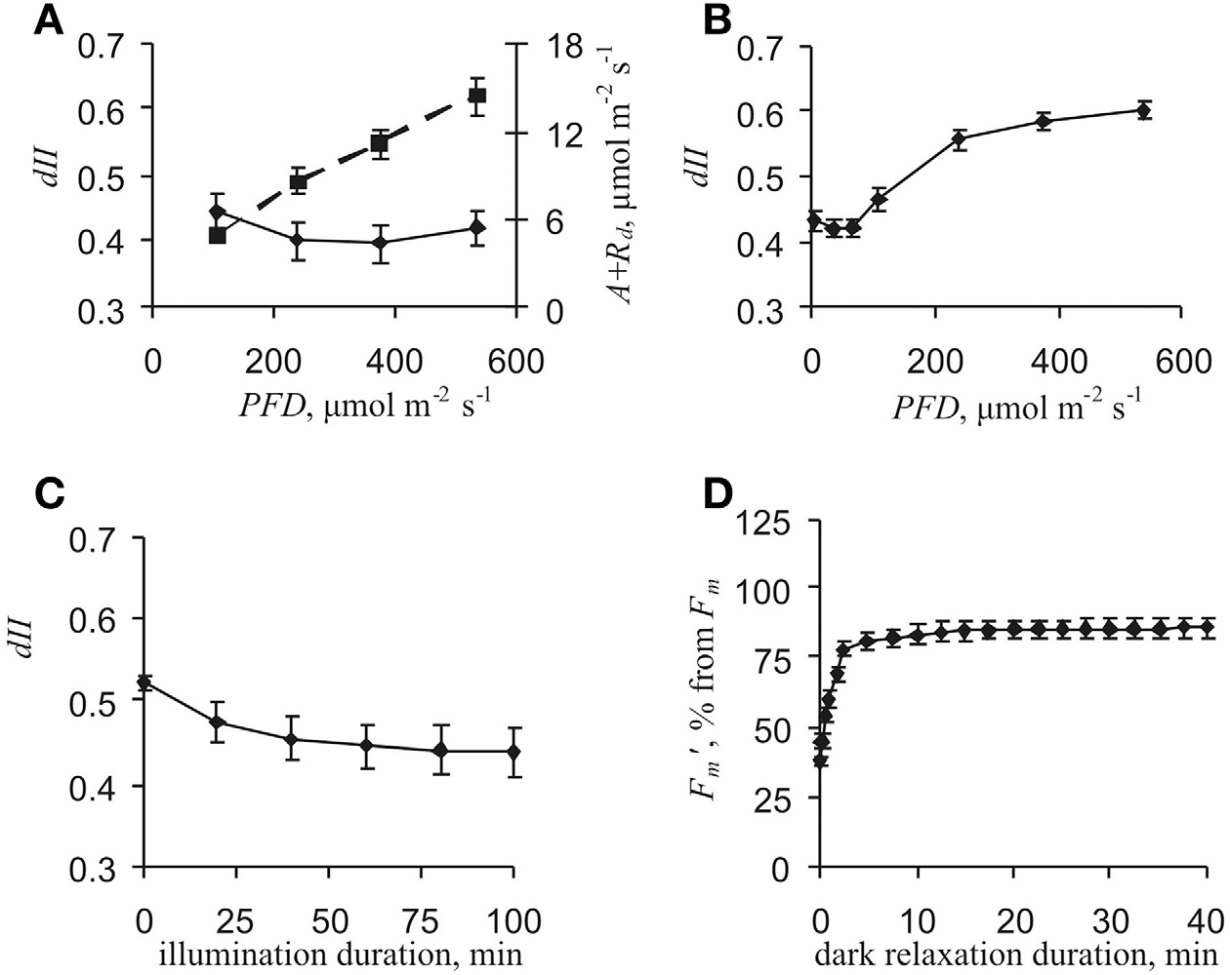
**Dependencies of photosynthetic parameters on light intensity and duration of illumination by actinic light and darkness. (A)** dependencies of *dII* and *A* + *R*_*d*_ on light intensity after 1 h control actinic light. Values for *dII* calculated using Equation (4) under external CO_2_ concentration of 2000 ppm (*n* = 7). **(B)** dependence of *dII* on light intensity after 1 h control actinic light. Value of *dII* calculated using equation (7) (*n* = 6). **(C)** dependence of *dII* on duration of control actinic light illumination. Value of *dII* calculated using Equation (7); actinic light intensity, 47 μmol·m^−2^·*s*^−1^ (*n* = 5). **(D)** dependence of *F*′_*m*_ on duration of darkness after actinic light illumination for 1 h.

Equation (6) was in good accordance with the works of Miyake et al. (2004); Miyake et al. (2005). Figure 2A shows *dII* calculated under different *PFD*s and an external [CO_2_] of 2000 ppm. This condition was electron transport limited because *A* + *R*_*d*_ depended on *PFD* in a linear manner. The value of *dII* varied from 0.40 to 0.44 and was not significantly dependent on light intensity (*p* > 0.05).

For the purpose of additional *dII* control, an alternative method for measuring *dII* was used (Huang et al., 2012) that was simpler than the previous method. It is known that plants have slight cyclic electron flow under low light intensity and that flow magnitude increases with increasing *PFD* (Miyake et al., 2005; Joliot and Joliot, 2006; Huang et al., 2011; Zivcak et al., 2013). Therefore, *EF(PSI)* approximately equals *EF(PSII)* under low light condition (Huang et al., 2012). Taking into account that *EF*(*PSI*) = *EF*(*PSII*) and using Equations (1) and (2), Equation (7) was deduced:

(7) $$dII=\frac{1}{\phi_{PSII}/\phi_{PSI}+1}.$$

Figure 2B shows that *dII* equaled ~0.42 under low light conditions (*PFD* ≤ 65 μmol·m^−2^·s^−1^). Increases in *dII* were observed under light intensity equaling 108 μmol·m^−2^·s^−1^ and greater. This increase probably reflected increased cyclic electron flow, i.e., *EF*(*PSI*) ≠ *EF*(*PSII*) under moderate and high light conditions. Thus, Equation (7) could also be used for *dII* calculation under low actinic light (≤65 μmol·m^−2^·s^−1^).

Values for *dII* calculated after a 1 h illumination by control actinic light (239 μmol·m^−2^·s^−1^) were in accordance with the time of VP induction. However, *dII* might have depended on the duration of actinic light illumination, which could have influenced results. Values of *dII* decreased from about 0.51–0.53 to ~0.41–0.46 with increased light duration (*t*_1/2_ = 20 min, Figure 2C), but it was essentially unchanged from the 60th to 100th min; i.e., *dII* was constant in the range of photosynthetic response investigated here.

Initial *dII* changes could have been connected with a state-transition and/or PSII damage. State-transition relaxation duration is from minutes to tens of minutes and damage relaxation time in hours (Maxwell and Johnson, 2000; Müller et al., 2001). As a result, state-transition might have been altered under VP rather than with PSII damage. Analysis of *F*′_*m*_ relaxation kinetics in darkness after 1 h of actinic light illumination showed that there were insignificant changes in this parameter from the 5th to 40th min (Figure 2D); i.e., there was no essential state-transition under these experimental conditions. In addition, this result supported the observed *dII* stability in the time range of VP-induced photosynthetic responses. Thus, taking into account these results, a *dII* value of 0.42 was used in the present work.

Far red light, which is absorbed predominantly by PSI, was used as actinic light in an individual series of experiments. In this case, *EF(PSII)* was also described by Equation (1); however, far red light absorption by PSII, which is low (Joliot and Johnson, 2011), was assumed equal to zero and *PFD* to be small (5 μmol·m^−2^·s^−1^, measuring light). Here, *EF(PSI)* was described by Equation (8):

(8) $$EF(PSI)=p\times \left(1-dII\right)\times \phi_{PSI}\times \left(PFD+FRFD\times \delta \right),$$

where *FRFD* was the far red light flux density, δ the ratio of far red light to actinic light absorption by leaf (730 and 460 nm, respectively), *PFD* = 5 μmol·m^−2^·s^−1^, and δ has been calculated from green leaf absorption spectra (Hogewoning et al., 2012) and equaled ~0.12.

Relative *EF(C)* was also used in analyses, as the percentage of cyclic electron flow in the total flow. Relative *EF(C)* was calculated using Equation (9):

(9) $$relative\;EF(C)=\frac{EF(C)}{EF(C)+EF(PSII)}\times 100\%.$$

### Statistics

Each series of experiments comprised 5–17 measurements, with each measurement performed on a separate plant. Representative records, obtained for individual measurements, mean values, standard errors, and correlation coefficients, are presented in the accompanying figures and tables. Significant differences in experiments were indicated according to the paired Student's *t*-test.

## Results

### Photosynthetic responses induced by variation potential

**Figure 3:**
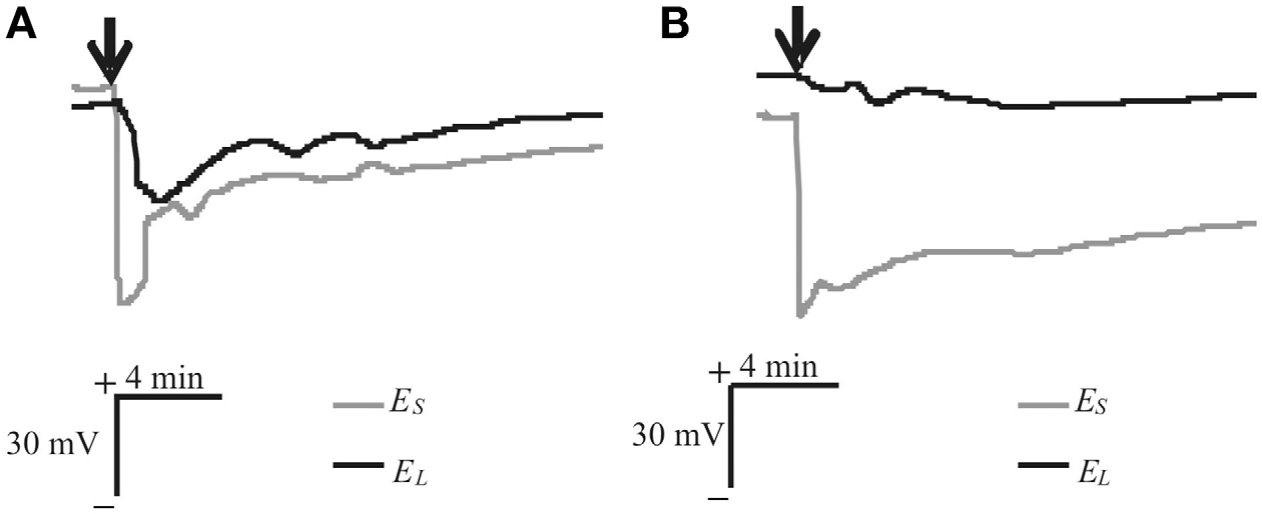
**Heat-induced changes in surface electrical potentials of steam and leaf. (A)** VP propagated into leaf (*n* = 17). **(B)** Only electrical reactions of small amplitude (<15 mV) propagated into leaf (*n* = 4). VP induced by heating tip of another leaf (arrow), and *E*_*S*_ and *E*_*L*_, changes in electrical potential measured by electrodes on stem and lamina, respectively.

Local heating of a leaf induced VP propagation through the stem (Figure 3A). The signal amplitude was 40–90 mV, the duration wide-ranging (from 5 to 60 min), and the profile varied. In the most experiments (~80%), VP propagated into leaf lamina with an amplitude of 20–75 mV, with propagation velocities between steam and leaf at 0.02–0.20 cm·s^−1^. In some experiments (~20%), only electrical reactions with small amplitude (<15 mV) were observed in lamina (Figure 3B).

**Figure 4:**
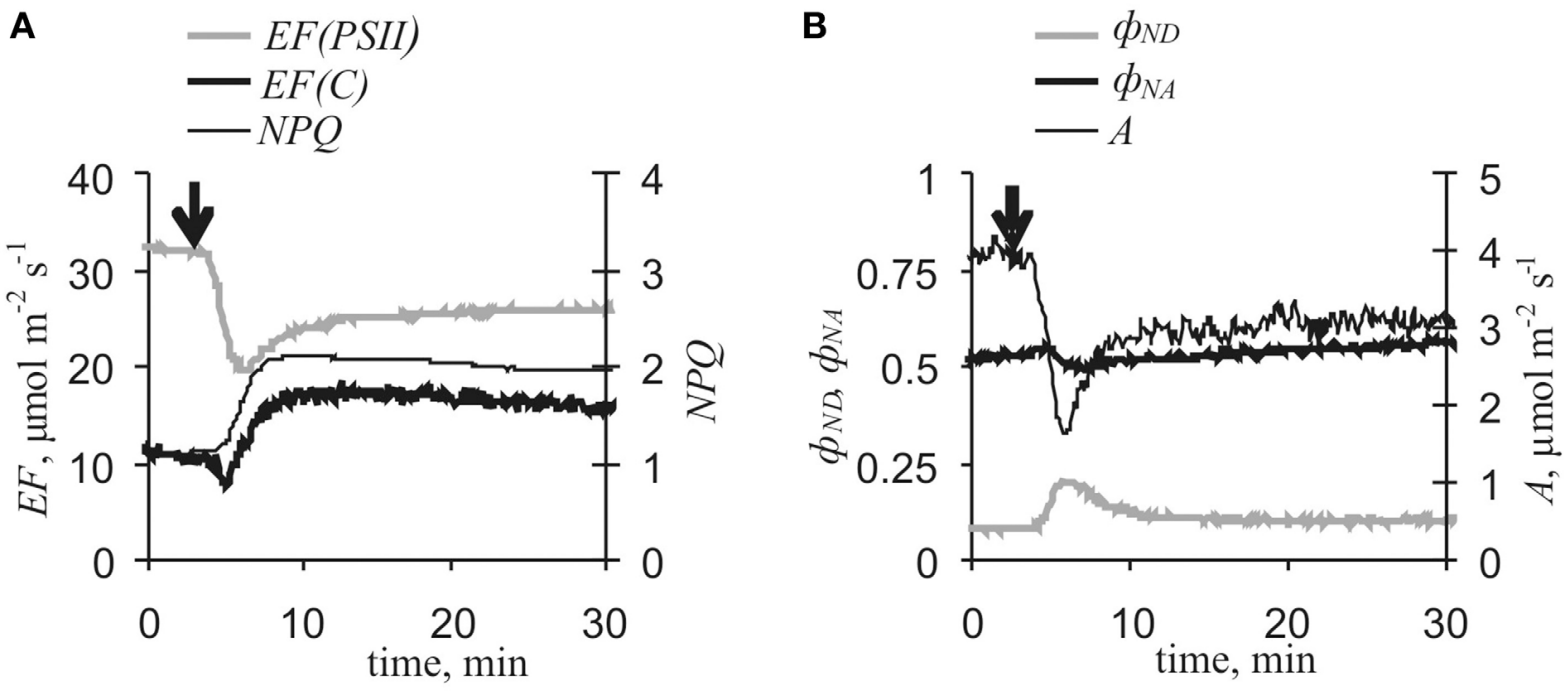
**Variation potential-induced changes in photosynthetic light stage parameters and CO_2_ assimilation under control conditions (*n* = 17). (A)** *EF(PSII), EF(C)* and *NPQ*. **(B)** ϕ_*ND*_, ϕ_*NA*_, and *A*. Arrow, VP induction by local heating.

Figure 4 and Table 1 show that VP reduced CO_2_ assimilation rates and electron flows through PSII, increased *NPQ* and ϕ_*ND*_, and weakly decreased ϕ_*NA*_. The response of *EF(C)* comprised two stages: fast inactivation of cyclic flow (*EF(C)*_*min*_ – *EF(C)*_*initial*_) and a following slow activation (*EF(C)*_*max*_ - *EF(C)*_*min*_). Extremes of *EF(PSII)* decrease, *EF(C)* fast inactivation, and *EF(C)* slow activation were observed at 2.8 ± 0.3, 1.0 ± 0.1 and 5.9 ± 0.3 min, respectively, after the start of photosynthetic responses. Fast inactivation and slow activation were probably independent of each other under control conditions because the correlation coefficient (*r*) between *EF(C)*_*min*_ - *EF(C)*_*initial*_ and *EF(C)*_*max*_ - *EF(C)*_*min*_ was −0.07 (*p* > 0.05). If only electrical reactions with small amplitudes (<15 mV) were observed in leaf lamina, a photosynthetic response was not developed.

**Table 1:** **Photosynthetic parameters and changes induced by VP and [CO_2_]-lowering**.

	**VP under [CO_2_] = 360 ppm**	**[CO_2_]- lowering to 10–15 ppm**	**[CO_2_]- lowering to 150 ppm**	**VP under [CO_2_] = 10–15 ppm**
*n*	17	9	10	11
*A* decrease, μmol·m^−2^·s^−1^	−1.95 ± 0.19^*^	−4.08 ± 0.3^*^	−1.91 ± 0.20^*^	−1.03 ± 0.15^*^
*EF(PSII)*_*initial*_, μmol·m^−2^·s^−1^	30.1 ± 1.2	33.1 ± 2.2	34.3 ± 1.3	13.7 ± 1.4
*EF(PSII)*_*min*_, μmol·m^−2^·s^−1^	23.6 ± 1.1^*^	13.5 ± 1.6^*^	25.9 ± 1.1^*^	8.1 ± 1.0^*^
*EF(PSII)*_*min*_- *EF(PSII)*_*initial*_, μmol·m^−2^·s^−1^	−6.5 ± 0.7^*^	−19.7 ± 1.3^*^	−8.3 ± 0.9^*^	−5.5 ± 0.8^*^
*EF(C)*_*initial*_, μmol·m^−2^·s^−1^	11.1 ± 1.4	11.7 ± 2.3	11.3 ± 1.9	18.1 ± 1.2
*EF(C)*_*min*_, μmol·m^−2^·s^−1^	8.6 ± 1.2^*^	11.7 ± 2.3	11.1 ± 1.8	15.0 ± 1.2^*^
*EF(C)*_*max*_, μmol·m^−2^·s^−1^	14.4 ± 1.1^*^	20.0 ± 1.6^*^	15.3 ± 2.1^*^	18.6 ± 1.3
*EF(C)*_*min*_- *EF(C)*_*initial*_, μmol·m^−2^·s^−1^	−2.6 ± 0.3^*^	0	−0.2 ± 0.1	−3.1 ± 0.4^*^
*EF(C)*_*max*_- *EF(C)*_*min*_, μmol·m^−2^·s^−1^	5.8 ± 0.4^*^	8.3 ± 1.1^*^	4.2 ± 0.5^*^	3.6 ± 0.5^*^
*NPQ*_*initial*_	1.16 ± 0.05	1.25 ± 0.14	1.29 ± 0.09	2.76 ± 0.14
*NPQ*_*max*_	1.97 ± 0.08^*^	2.69 ± 0.17^*^	1.86 ± 0.14^*^	3.44 ± 0.16^*^
*NPQ*_*max*_- *NPQ*_*initial*_	0.81 ± 0.06^*^	1.34 ± 0.11^*^	0.57 ± 0.08^*^	0.68 ± 0.13^*^
ϕ_*ND initial*_	0.081 ± 0.009	0.071 ± 0.016	0.048 ± 0.006	0.202 ± 0.025
ϕ_*ND max*_	0.142 ± 0.011^*^	0.182 ± 0.014^*^	0.100 ± 0.012^*^	0.253 ± 0.021^*^
ϕ_*ND max*_- ϕ_*ND initial*_	0.061 ± 0.008^*^	0.111 ± 0.010^*^	0.035 ± 0.006^*^	0.051 ± 0.009^*^
ϕ_*NA initial*_	0.584 ± 0.015	0.559 ± 0.027	0.563 ± 0.027	0.536 ± 0.028
ϕ_*NA min*_	0.572 ± 0.014^*^	0.531 ± 0.025^*^	0.551 ± 0.028^*^	0.549 ± 0.026
ϕ_*NA min*_- ϕ_*NA initial*_	−0.012 ± 0.002^*^	−0.029 ± 0.007^*^	−0.012 ± 0.003^*^	0.013 ± 0.006

* *p < 0.05 compared with control, paired Student t-test*.

Responses in *EF(C)* might have been connected with VP-induced changes in *dII*. The method for *dII* calculation by Huang et al. (2012) could not be used for plants that were stressed after local heating. However, *dII* changes must have modified *F*′_*m*_ under dark conditions. VP's influence on *F*′_*m*_ without actinic light was investigated here. It was shown that VP induced only small decrease of *F*′_*m*_ (3 ± 1%, *n* = 5), i.e., VP weakly influence dII.

**Figure 5:**
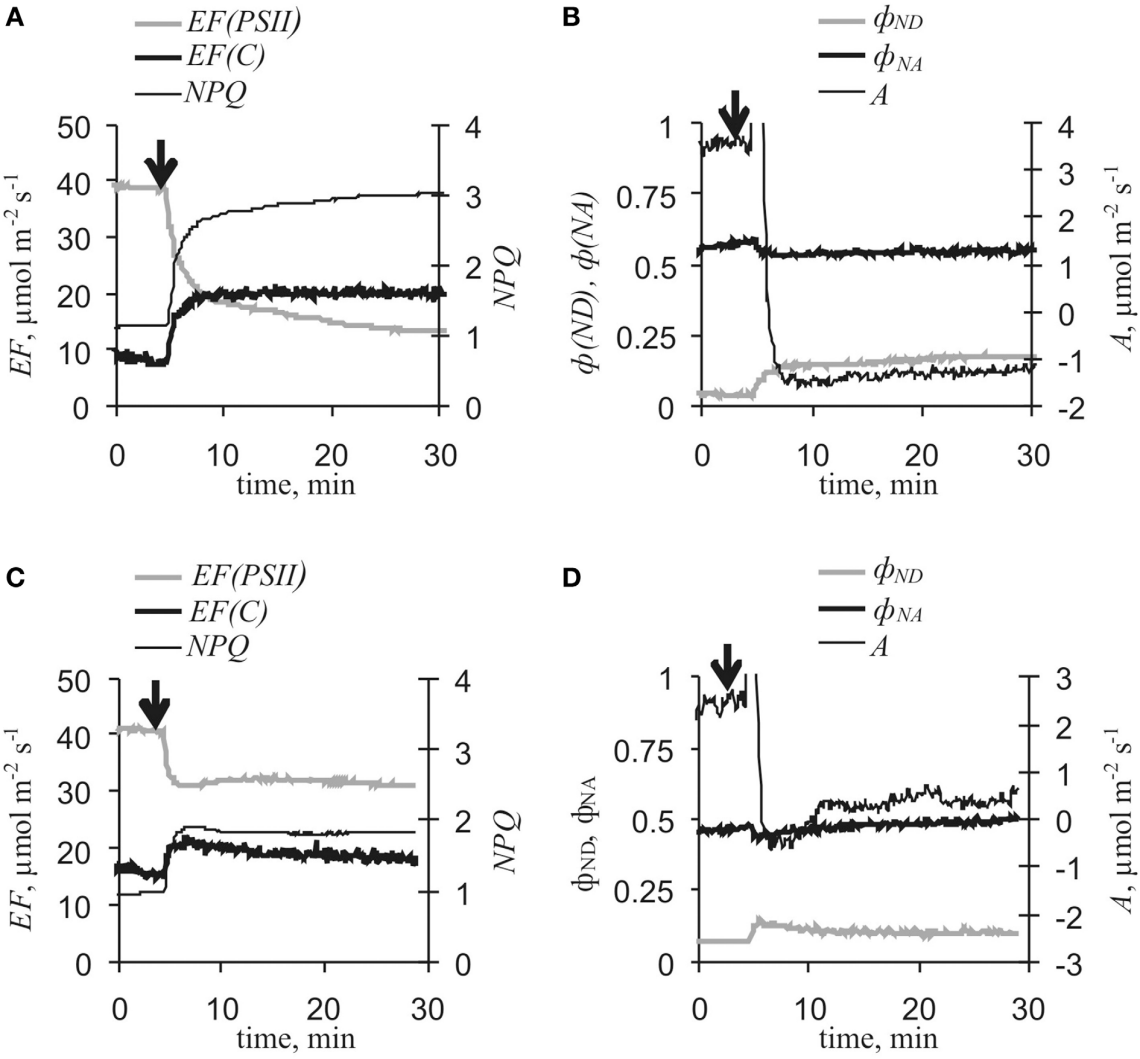
**[CO_2_]-lowering-induced changes in photosynthetic light stage parameters and CO_2_ assimilation (*n* = 9–10). (A)** *EF(PSII), EF(C)* and *NPQ* under [CO_2_]-lowering to 10–15 ppm. **(B)** ϕ_*ND*_, ϕ_*NA*_, and *A* under [CO_2_]-lowering to 10–15 ppm. **(C)** *EF(PSII), EF(C)* and *NPQ* under [CO_2_]-lowering to 150 ppm. **(D)** ϕ_*ND*_, ϕ_*NA*_, and *A* under [CO_2_]-lowering to 150 ppm. Initial [CO_2_] was 360 ppm. Arrow, start of [CO_2_]-lowering.

**Figure 6:**
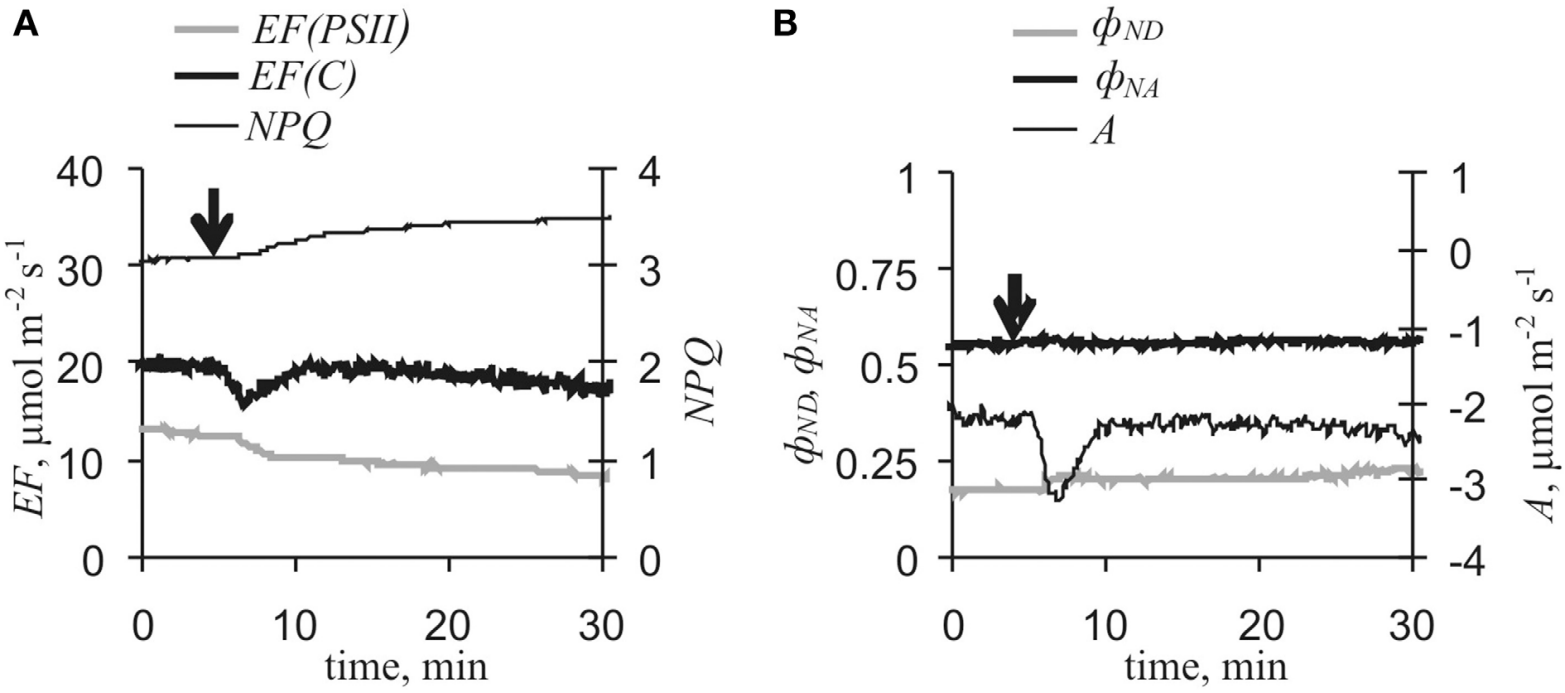
**Variation potential-induced changes in photosynthetic light stage parameters and CO_2_ assimilation under low [CO_2_] conditions (*n* = 11). (A)** *EF(PSII), EF(C)* and *NPQ*. **(B)** ϕ_*ND*_, ϕ_*NA*_, and *A*. [CO_2_] was 10–15 ppm. Arrow, VP induction by local heating.

Inactivation of the photosynthetic dark stage is an initial process of VP-induced photosynthetic responses in pea (Sukhov et al., 2014a,b) and geranium (Sukhov et al., 2012). The present results showed that VP-induced *A* and *EF(PSII)* decreases were strongly correlated (*r* = 0.78, *p* < 0.001). Artificial reduction of dark stage activity through lowering of external [CO_2_] decreased *EF(PSII)* and increased *EF(C)*, ϕ_*ND*_ and *NPQ* (Figure 5), but fast inactivation of cyclic flow was absent. Moreover, VP-induced decreases in *A* and *EF(PSII)*, increases in ϕ_*ND*_ and *NPQ*, and slow activation of *EF(C)* were collectively smaller under low [CO_2_] conditions (10–15 ppm) than under control conditions (Figure 6, Table 1). In particular, maximum cyclic flow after VP was not distinguishable from the flow before electrical signal propagation under these conditions. However, fast inactivation of cyclic flow induced by VP under low [CO_2_] was not significantly different from the controls. It should be noted that the correlation coefficient between EF(C)_min_ - EF(C)_initial_ and EF(C)_max_ - EF(C)_min_ was −0.81 (*p* < 0.01) under low [CO_2_].

### Correlation analysis of the mechanism of VP-induced cyclic electron flow changes

Changes in *EF(PSII)* and *EF(C)* might be different stages of united VP-induced photosynthetic response. This hypothesis was tested by analysis that revealed correlations between photosynthetic parameter changes (Table 2). There were strong connections between VP or [CO_2_]-lowering-induced reduction of electron flow through PSII and slow activation of cyclic electron flow under control initial conditions. Correlation between decreases in *EF(PSII)* and fast inactivation of *EF(C)* was insignificant. Under low [CO_2_], VP-induced responses of *EF(PSII)* and *EF(C)* were weakly connected with each other.

**Table 2:** **Correlation coefficients between changes in photosynthetic parameters induced by VP and [CO_2_]-lowering**.

**Parameters**	**VP under [CO_2_] = 360 ppm**	**[CO_2_]- lowering to 10–15 or 150 ppm**	**VP under [CO_2_] = 10–15 ppm**
*n*	17	19	11
*EF(PSII)*_*min*_- *EF(PSII)*_*initial*_ and *EF(C)*_*min*_ - *EF(C)*_*initial*_	0.11	−	−0.15
*EF(PSII)*_*min*_- *EF(PSII)*_*initial*_ and *EF(C)*_*max*_- *EF(C)*_*min*_	−0.89^*^	−0.85^*^	−0.04
*EF(PSII)*_*min*_- *EF(PSII)*_*initial*_ and ϕ_*ND max*_ - ϕ_*ND initial*_	−0.90^*^	−0.90^*^	−0.73
ϕ_*ND max*_ - ϕ_*ND initial*_ and *EF(C)*_*max*_- *EF(C)*_*min*_	0.80^*^	0.77^*^	−0.20

* *Correlation coefficient is significant (p < 0.05), Student t-test*.

The connection between *EF(PSII)* decrease and slow *EF(C)* activation might have been caused by changes in ϕ_*ND*_. Really, correlation analysis showed that electron flow reductions through PSII induced by VP or [CO_2_]-lowering was strongly correlated with increases in ϕ_*ND*_ (Table 2). Conversely, ϕ_*ND*_ increases were correlated with slow cyclic electron flow activation under control initial conditions, but the correlation coefficient was insignificant for VP-induced responses under initial low [CO_2_].

### VP-induced cyclic electron flow changes under far red light

**Figure 7:**
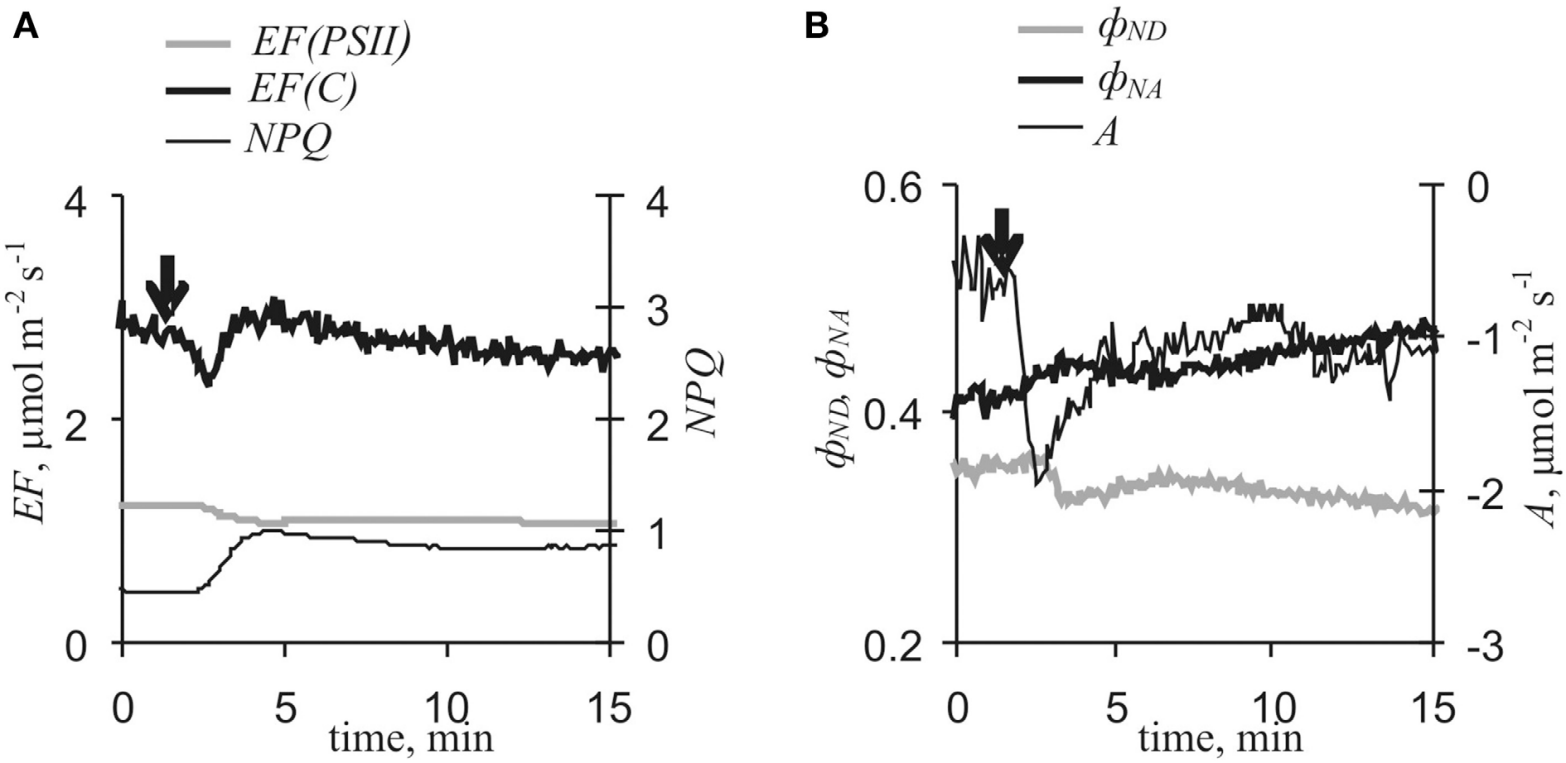
**Variation potential-induced changes in photosynthetic light stage parameters and CO_2_ assimilation under far red light conditions (*n* = 9). (A)** *EF(PSII), EF(C)* and *NPQ*. **(B)** ϕ_*ND*_, ϕ_*NA*_, and *A*. Arrow, VP induction by local heating.

Far red light selectively activates PSI and is widely used for cyclic electron flow investigations (Joliot and Johnson, 2011). Here, far red light conditions were used for more detailed investigation of VP's influence on cyclic electron flow, and Figure 7 and Table 3 show VP-induced photosynthetic responses under far red light. VP decreased *A, EF(PSII)*, and ϕ_*ND*_ and increased ϕ_*NA*_ and *NPQ*. VP-induced changes in *EF(C)* included two stages as described above, inactivation and subsequent activation. Both activation and inactivation were weakly connected with decreased electron flow through PSII. Correlation between *EF(PSII)* and ϕ_*ND*_ decreases was also insignificant. Conversely, slow *EF(C)* activation was strongly correlated with decreased ϕ_*ND*_. Connections of changes in ϕ_*NA*_ with decreases in *EF(PSII)* and increases in *EF(C)* were insignificant (data not shown). It should be noted that there was a tenuous connection between *EF(C)*_*min*_ - *EF(C)*_*initial*_ and *EF(C)*_*max*_ - *EF(C)*_*min*_ under far red light conditions (*r* = −0.65, *p* = 0.06).

**Table 3:** **Photosynthetic parameters and changes induced by VP and correlation coefficients under far red light conditions**.

**PARAMETERS AND THEIR CHANGES**				
*A* decrease, μmol·m^−2^·s^−1^	−1.08 ± 0.10^*^	ϕ_*ND initial*_		0.337 ± 0.013
*EF(PSII)*_*initial*_, μmol·m^−2^·s^−1^	1.24 ± 0.02	ϕ_*ND min*_		0.295 ± 0.014^*^
*EF(PSII)*_*min*_, μmol·m^−2^·s^−1^	1.11 ± 0.03^*^	ϕ_*ND min*_ - ϕ_*ND initial*_		−0.042 ± 0.005^*^
*EF(PSII)*_*min*_- *EF(PSII)*_*initial*_, μmol·m^−2^·s^−1^	−0.13 ± 0.02^*^	ϕ_*NA initial*_		0.420 ± 0.012
*EF(C)*_*initial*_, μmol·m^−2^·s^−1^	2.98 ± 0.06	ϕ_*NA max*_		0.447 ± 0.013^*^
*EF(C)*_*min*_, μmol·m^−2^·s^−1^	2.60 ± 0.07^*^	ϕ_*NA max*_ - ϕ_*NA initial*_		0.027 ± 0.002^*^
*EF(C)*_*max*_, μmol·m^−2^·s^−1^	3.27 ± 0.10^*^	*NPQ*_*initial*_		0.52 ± 0.03
*EF(C)*_*min*_- *EF(C)*_*initial*_, μmol·m^−2^·s^−1^	−0.38 ± 0.04^*^	*NPQ*_*max*_		0.99 ± 0.11^*^
*EF(C)*_*max*_- *EF(C)*_*min*_, μmol·m^−2^·s^−1^	0.66 ± 0.08^*^	*NPQ*_*max*_- *NPQ*_*initial*_		0.47 ± 0.08^*^
**CORRELATION COEFFICIENTS**				
*EF(PSII)*_*min*_- *EF(PSII)*_*initial*_	0.35	*EF(PSII)*_*min*_- *EF(PSII)*_*initial*_		0.46
and		and		
*EF(C)*_*min*_- *EF(C)*_*initial*_		ϕ_*ND max*_ - ϕ_*ND initial*_		
*EF(PSII)*_*min*_- *EF(PSII)*_*initial*_	−0.53	ϕ_*ND max*_ - ϕ_*ND initial*_		−0.86^#^
and		and		
*EF(C)*_*max*_- *EF(C)*_*min*_		*EF(C)*_*max*_- *EF(C)*_*min*_		

* *p < 0.05 compared with control, paired Student t-test (n = 9)*.

# *Correlation coefficient is significant (p < 0.05), Student t-test*.

### VP- and [CO_2_]-lowering-induced increases in relative cyclic electron flow

Examination of changes in relative cyclic electron flow induced by VP or [CO_2_]-lowering showed that, with VP under control, low [CO_2_], and far red light conditions as well as decreased [CO_2_] alone induced increases in relative electron flow (Table 4). This effect was observed even if the absolute *EF(C)* showed no changes (photosynthetic response induced by VP under low [CO_2_]).

**Table 4:** **Relative cyclic electron flow and changes induced by VP and [CO_2_]-lowering**.

	**VP**	**[CO_2_]- lowering to 10–15 ppm**	**[CO_2_]- lowering to 150 ppm**	**VP under [CO_2_] = 10-15 ppm**	**VP under far red light**
Relative *EF(C)*_*initial*_, %	26.6 ± 3.2	25.5 ± 4.9	23.5 ± 3.3	57.2 ± 3.8	70.7 ± 0.5
Relative *EF(C)*_*max*_, %	37.9 ± 2.7^*^	59.9 ± 3.5^*^	35.8 ± 3.7^*^	69.5 ± 3.0^*^	74.5 ± 0.9^*^
Change in relative *EF(C), %*	11.3 ± 1.3^*^	34.5 ± 3.0^*^	12.3 ± 1.0^*^	12.3 ± 1.6^*^	3.9 ± 0.6^*^

* *p < 0.05 compared with control, paired Student t-test (n = 9–17)*.

**Figure 8:**
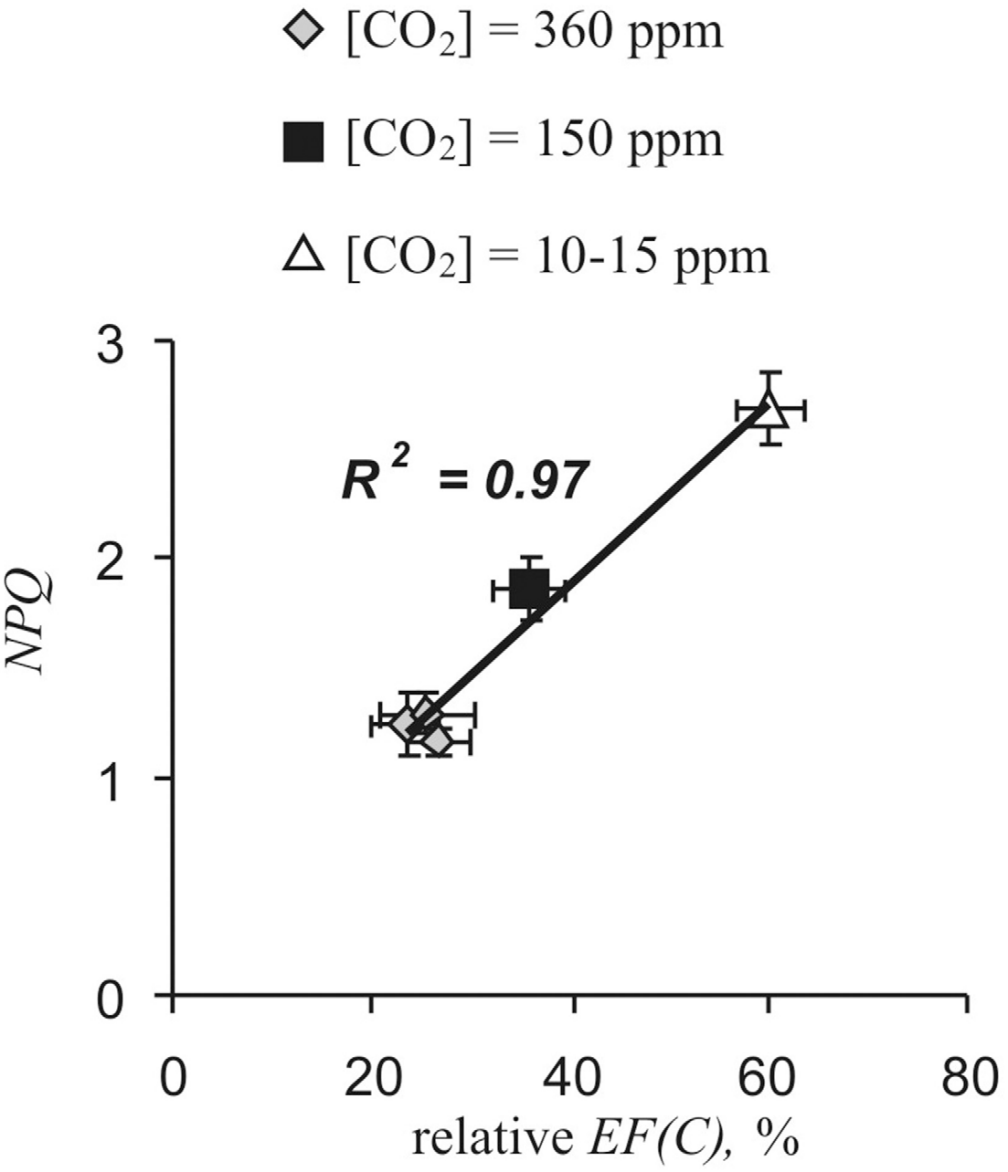
**Fluorescence non-photochemical quenching (*NPQ*) was plotted against relative cyclic electron flow [relative *EF(C)*] under different CO_2_ concentrations**. Relative *EF(C)* and NPQ were taken from the Tables 1, 4 (unheated plants).

Stimulation of NPQ is mechanism of cyclic electron flow influences on plant resistance to stressors (Zhang and Sharkey, 2009; Joliot and Johnson, 2011). *NPQ* has been plotted against *relative EF(C)* under different CO_2_ concentrations (Figure 8). *EF(C)* and NPQ were taken from the Tables 1, 4 (unheated plants). Figure 8 shows that *EF(C)* and NPQ were linearly connected.

Connection between relative cyclic electron flow and fluorescence non-photochemical quenching were observed under control conditions too. Correlation coefficient between *EF(C)* and NPQ was 0.64 (*n* = 36. *p* < 0.001).

## Discussion

Electrical signals can inactivate photosynthesis in plants (Koziolek et al., 2004; Krupenina and Bulychev, 2007; Grams et al., 2009; Pavlovič et al., 2011; Sukhov et al., 2012, 2013a, 2014a,b). In particular, VP reduces ϕ_*PSI*_, ϕ_*PSII*_, and A, and increases *NPQ* in pea (Sukhov et al., 2014a,b). Inactivation of the photosynthetic dark stage appears to be the initiator of photosynthetic responses induced by AP (Pavlovič et al., 2011) and VP (Sukhov et al., 2012, 2014a,b). However, direct influence of electrical signals on PSI (Sukhov et al., 2012) and PSII (Pavlovič et al., 2011; Sukhov et al., 2014a,b) is also observed.

Our results showed that VP, propagating into a leaf (Figure 3), induced changes in photosynthetic electron flows (Figure 4). VP-induced *EF(PSII)* decreases were in good accordance with data regarding ϕ_*PSII*_ decreases caused by electrical signals (see above). However, changes in *EF(C)*, which included fast cyclic electron flow inactivation and its subsequent slow activation, were not previously shown and required detailed analysis.

Here, *dII* was shown to be stable in the range of photosynthetic response investigated (Figure 2C), and a state-transition was insignificant under these experimental conditions (Figure 2D). Also, VP weakly influenced *F*_*m*_ without actinic light and, therefore, slightly changed *dII*. These results revealed that the *EF(C)* response was not connected with *dII* changes.

Slow cyclic electron transport activation induced by VP and [CO_2_]-lowering was observed to be connected with decreased *EF(PSII)* and that this connection was mediated by increased ϕ_*ND*_ (Table 2). Low activity of the photosynthetic dark stage and noncyclic electron flow are known to be accompanied by high *EF(C)* (Joliot and Joliot, 2006) as well as ϕ_*ND*_ value is positively correlated with cyclic flow magnitude (Munekage et al., 2002, 2004; Zivcak et al., 2013); however, the mechanisms of these connections remain unclear.

**Figure 9:**
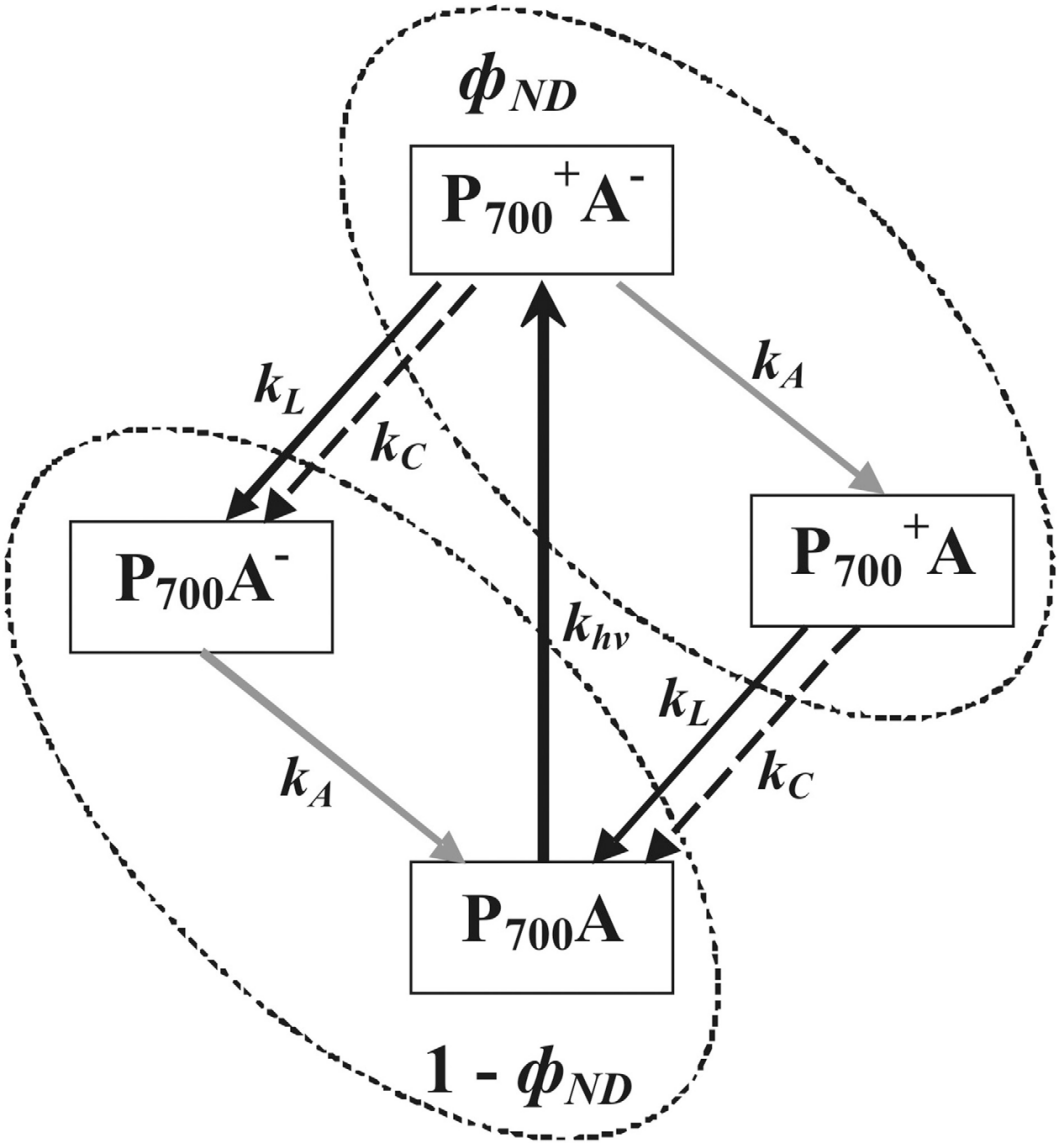
**A simple schema of PSI states, based on Klughammer and Schreiber (1994) and Vredenberg and Bulychev (2010)**. P^+^_700_ and P_700_, oxidized and reduced forms of primary electron donor in PSI, respectively; A and A^−^, oxidized and reduced forms PSI acceptor, respectively; parameter *k*_*hv*_ = γ × *PFD*× *p* × (1 − *dII*), velocity constant of light-dependant P_700_ oxidation, with γ, proportionality coefficient; parameters *k*_*L*_ = *k*_*PC*_ × [*PC*^−^ ]_*L*_ and *k*_*C*_ = *k*_*PC*_ × [*PC*^−^]_*C*_, velocity constants of electron flow through PS II (mainly noncyclic) and cyclic electron flow, respectively; *k*_*PC*_, velocity constant of plastocyanin oxidation by P^+^_700_; [PC^−^]_*L*_, concentration of reduced plastocyanin, participating in flow through PS II; [PC^−^]_*C*_, concentration of reduced plastocyanin, participating in cyclic flow; *k*_*A*_, velocity constant of PSI acceptor side oxidation being connected with cyclic, noncyclic and pseudocyclic flows; ϕ_*ND*_, portion of P^+^_700_A^−^ and P^+^_700_A (Klughammer and Schreiber, 2008); and 1 - ϕ_*ND*_, portion of P_700_A^−^ and P_700_A.

The simplest schema of PSI, based on Klughammer and Schreiber (1994) and Vredenberg and Bulychev (2010) and including P_700_ oxidation and P^+^_700_ reduction, was used here for analysis of photosynthetic response mechanisms (Figure 9). Using this schema, *EF(PSII)* and *EF(C)* were described by Equations (10) and (11):

(10) $$EF(PSII)=k_{L}\times \phi_{ND}$$

(11) $$EF(C)=k_{C}\times \phi_{ND}$$

There are two possible mechanisms for the observed connection between increases in ϕ_*ND*_ and *EF(C)* (Table 2). (i) Increased *k*_*C*_ is the main mechanism for increasing cyclic electron flow induced by VP or [CO_2_]-lowering under control conditions. This can be caused by activation of any stage of cyclic electron flow, with the exception of P^+^_700_ reduction, and increased concentrations of reduced plastocyanin. In this case, *k*_*C*_ increases contribute to transformation of P^+^_700_ into P_700_ and thereby lowers ϕ_*ND*_ (Figure 9). Thus, decreased ϕ_*ND*_, increased photosynthetic cyclic electron flow, and negative correlation between changes in *EF(C)* and ϕ_*ND*_ must appear that are contrary to the present experimental results (Figures 4, 5, Tables 1, 2). (ii) Decreases in *k*_*L*_ suppress transformation of P^+^_700_ into P_700_ and/or increases in *k*_*hv*_ activate conversion of P_700_ into P^+^_700_ that then increases ϕ_*ND*_ (Figure 9). According to Equation (11), increased ϕ_*ND*_ must activate cyclic electron flow. As a result, ϕ_*ND*_ and *EF(C)* increase and, in this case, a positive correlation between changes in *EF(C)* and ϕ_*ND*_ should be observed. The second variant was in perfect accordance with the present experimental results. Thus, the following chain of events is supposed: VP → … → ϕ_*ND*_ increase → *EF(C)* growth.

Similar analysis can be employed for examining connections between increased ϕ_*ND*_ and reduced *EF(PSII)* (Figures 4, 5, Tables 1, 2). The positive influence of *k*_*C*_ changes was not probable because increased *k*_*C*_ decreases ϕ_*ND*_(see above) and decreased *k*_*C*_ decreases *EF(C)* (Equation 11). Increased *k*_*hv*_ appeared to increase ϕ_*ND*_(Figure 9); however, if ϕ_*ND*_ increased and *k*_*L*_ was not changed, then *EF(PSII)* must increase (Equation 10), which is contrary to experimental results. Moreover, the main reason for *k*_*hv*_ changes was modification of *dII*, but *dII* was probably not affected by VP. Alternatively, decreased *k*_*L*_ suppressed transformation of P^+^_700_ into P_700_ and increased ϕ_*ND*_ (Figure 9), while also lowering *EF(PSII)* (Equation 10). The *k*_*L*_ decrease could have been caused by inactivation of any stage of noncyclic electron transport, which preceded PSI, and which induced decreased concentrations of reduced plastocyanin. This last variant was in a good accordance with the experimental results obtained here (decreased *EF(PSII)*, increased ϕ_*ND*_, and negative correlation between changes in *EF(PSII)* and ϕ_*ND*_). Decreased *k*_*L*_ reflected decreased electron flow from PSII. Thus, the chain of events was extended to yield: VP→ … → *EF(PSII)* decrease → ϕ_*ND*_ increase → *EF(C)* growth.

The present results indicate that decreased *EF(PSII)* reflects VP-induced lowering of ϕ_*PSII*_ (Figure 4, Table 1). According to published data (Pavlovič et al., 2011; Sukhov et al., 2012, 2014a,b) and the present results, this decrease in *EF(PSII)* was mainly induced by photosynthetic dark stage inactivation. In support of this conclusion, changes in *A* and *EF(PSII)* were strongly correlated and reduced CO_2_ assimilation rate induced by [CO_2_]-lowering decreased electron flow through PSII was similar to VP's effect. In addition, VP-induced *EF(PSII)* changes under low [CO_2_] conditions were smaller than changes under control conditions. However, VP-induced *EF(PSII)* decreases were not absent under low [CO_2_]. Considering that decreased *A* in these experiments (−1.08 ± 0.21 μmol·m^−2^·s^−1^) was indistinguishable from a VP-induced respiration response (−1.10 ± 0.20 μmol·m^−2^·s^−1^, Sukhov et al., 2014a), it was concluded that VP could also have suppressed electron flow through PSII without photosynthetic dark stage inactivation. Increased *NPQ* was a potential mechanism for VP's influence on PSII because its response is not dependent on electrical signal-induced decrease of CO_2_ assimilation (Sukhov et al., 2014a). As a result, the following chain of events was proposed here: VP → … → inactivation of photosynthetic dark stage and *NPQ* increase → *EF(PSII)* decrease → ϕ_*ND*_ increase → *EF(C)* increase.

**Figure 10:**
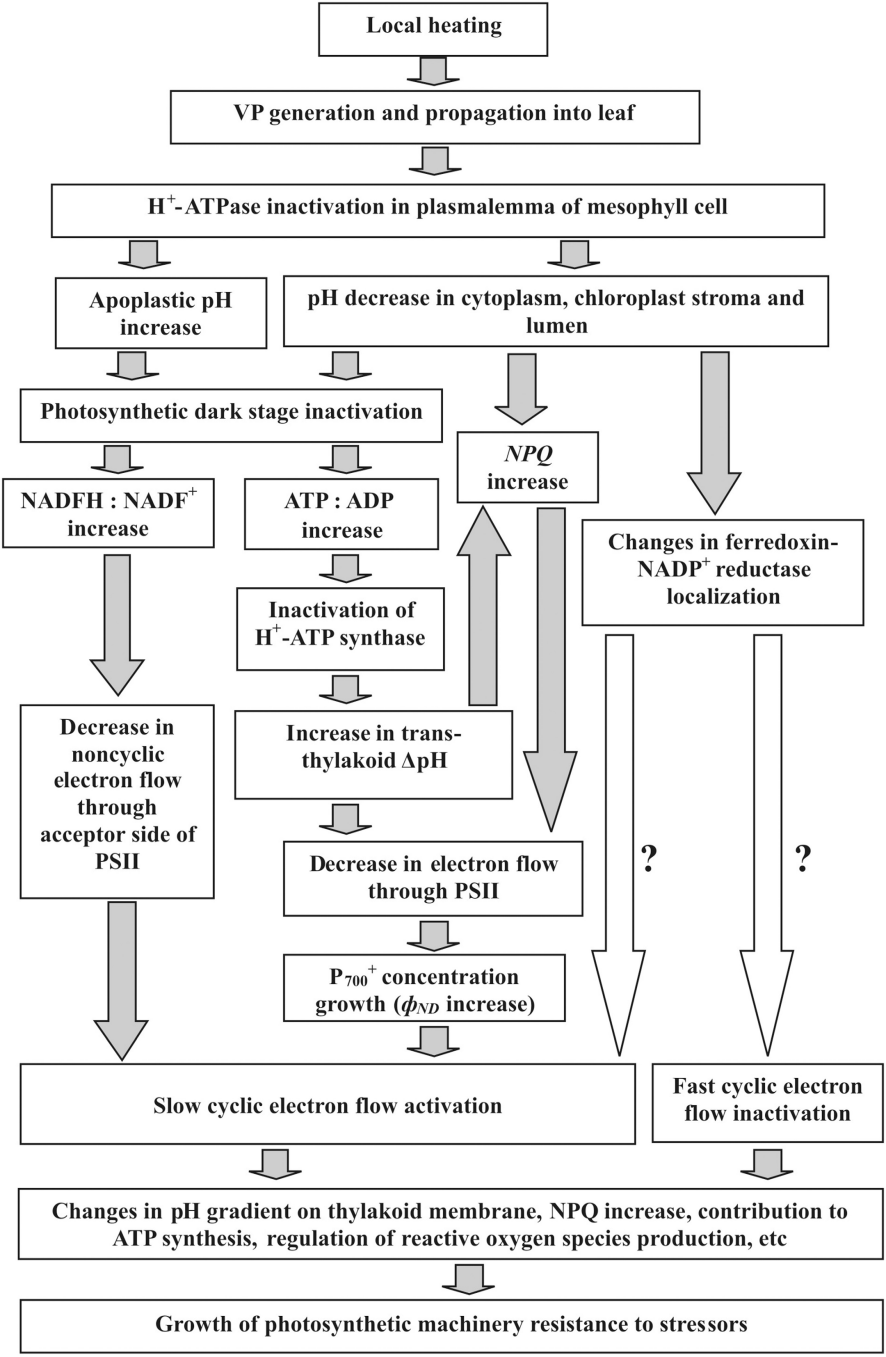
**Potential mechanisms of VP-induced cyclic electron flow responses and their functional role in higher plants**. Local heating induces VP, which propagates into leaves (our results, Sukhov et al., 2012, 2014a,b). VP generation connected with transient inactivation of H^+^-ATPase in plasmalemma (Stahlberg et al., 2006; Sukhov et al., 2013b). Alkalization of apoplast and acidification of cytoplasm (Grams et al., 2009; Sukhov et al., 2014a) are results of this inactivation. As chloroplast pH strongly depends on external medium in chloroplast suspensions (Werdan et al., 1975), cytoplasmic acidification can reduce pH in chloroplasts. Alkalization of apoplast and acidification of cytoplasm can suppress CO_2_ influx from apoplast to cytoplasm and stroma, and reduce Calvin cycle activity (Bulychev et al., 2001; Grams et al., 2009; Sukhov et al., 2014a). Acidification of stroma essentially suppresses photosynthesis (Werdan et al., 1975) that might be connected with pH-dependent Calvin cycle enzymes (Wolosiuk et al., 1993) and nonphotochemical energy dissipation in PSII (Müller et al., 2001). VP-induced photosynthetic dark stage suppression (our results, Sukhov et al., 2012, 2014a,b) inactivates H^+^-ATP synthase and increases trans-thylakoid ΔpH (Pavlovič et al., 2011) that reduces electron flow through PSII (Schönknecht et al., 1995) and additionally stimulates NPQ (Müller et al., 2001). Increase of nonphotochemical energy dissipation in PSII (our results, Sukhov et al., 2014a) also decreases electron flow through PSII (our results). In turn, this increases P^+^_700_ concentration and, thereby, stimulates cyclic electron flow (our results). Alternatively, photosynthetic dark stage inactivation may increase NADFH:NADF^+^ (Pavlovič et al., 2011) that can also intensify cyclic electron flow. There is an additional VP-induced slow cyclic electron flow activation, which is connected with fast cyclic flow inactivation, and is not affected by electron flow through PSII (our results). pH-Dependent changes in ferredoxin-NADP^+^ reductase localization (Alte et al., 2010; Benz et al., 2010) might participate in both additional activation and fast inactivation, because this enzyme possibly plays a role in cyclic electron flow (Joliot and Johnson, 2011). Finally, changes in cyclic electron flow might participate in electrical signal induced resistance of photosynthetic machinery to stressors (Retivin et al., 1999; Sukhov et al., 2014b). This might be because cyclic flow contributes to ATP synthesis, regulates oxygen species production, additionally increases *NPQ*, and keeps the PSI acceptor side oxidized, protecting it from damage (Joliot and Joliot, 2006; Rumeau et al., 2007; Zhang and Sharkey, 2009; Roach and Krieger-Liszkay, 2014).

VP-connected proton flux from apoplast to cytoplasm, stroma, and lumen is a possible mechanism for initial induction of a photosynthetic response, including decreased ϕ_*PSII*_ (Grams et al., 2009; Sukhov et al., 2014a). It is known that VP generation is connected with transient H^+^-ATPase inactivation and proton influx (Stahlberg et al., 2006; Sukhov et al., 2013b), which changes intra- and extracellular pH (Grams et al., 2009; Sukhov et al., 2014a). However, decreased intracellular pH can suppress PSII photosynthetic activity and induces *NPQ* (Grams et al., 2009; Bulychev et al., 2013a,b; Sukhov et al., 2013a, 2014a). Taking into account these facts, it can be proposed that VP → H^+^ influx → inactivation of photosynthetic dark stage and *NPQ* growth → *EF(PSII)* decrease → ϕ_*ND*_ increase → *EF(C)* growth. Figure 10 shows this possible mechanism of VP influence on cyclic electron flow in more detail.

It should be noted that photosynthetic dark stage inactivation can increase NADFH:NADF^+^ (Pavlovič et al., 2011) that decreases noncyclic electron flow through acceptor side of PSI and may stimulate cyclic electron flow. However, this process (decrease in k_*A*_ in Figure 9) induces increase in P_700_A^−^ (ϕ_*NA*_) that was not observed in experiments with varied CO_2_ concentrations (Table 1). Thus, change in NADFH:NADF^+^ in unlikely to be main mechanism of *EF(C)* growth, but it can play minor role in the process.

VP-induced responses under far red light indicated that another mechanism of cyclic electron flow activation, not connected with noncyclic flow changes, also participated in the photosynthetic response. In this case, *EF(C)* activation and decreased ϕ_*ND*_ were observed and correlation between these parameters was negative (Table 3). Considering Figure 9 and Equation (11), it was concluded that such effects could have been caused by increased *k*_*C*_. The mechanism of *EF(C)* activation was not clarified here, but the magnitude of the activation correlated with the magnitude of fast inactivation of cyclic electron flow; i.e., similar mechanisms for both processes were probable. It is known that pH decreases can change ferredoxin-NADP^+^ reductase localization (Alte et al., 2010; Benz et al., 2010); in addition, reductase possibly participates in cyclic electron flow (Joliot and Johnson, 2011). Considering this information, it was speculated that stromal pH changes influenced ferredoxin-NADP^+^ reductase localization and induced a two-stage *EF(C)* response, including inactivation and subsequent activation of cyclic flow (Figure 7). VP-induced inactivation of the acceptor side of PSI, which was not connected with decreased photosynthetic dark stage activity (Sukhov et al., 2012) and can caused by changes in ferredoxin-NADP^+^ reductase localization (Sukhov et al., 2014a), supported this hypothesis.

Thus, VP increased cyclic electron flow in an absolute (Tables 1, 3) and relative (Table 3) manner. A physiological role for this response could have been connected with increased photosynthetic machinery resistance to environmental stressors. It is known that cyclic electron flow can maintain a high proton gradient on thylakoid membrane and, thereby, contributes to ATP synthesis and *NPQ* increases (Zhang and Sharkey, 2009; Joliot and Johnson, 2011). Also, cyclic electron flow protects PSI and can regulate reactive oxygen species production by photosynthetic electron transfer chain (Rumeau et al., 2007; Roach and Krieger-Liszkay, 2014). It is known that electrical signals exert influence on the resistance of photosynthetic machinery to stressors in higher plants (Retivin et al., 1999; Sukhov et al., 2014b). In particular, VP increases PSI resistance to heating in pea (Sukhov et al., 2014b), and it was supposed here that VP-induced activation of cyclic electron flow participated in this increased resistance.

Stimulation of NPQ is important mechanism of cyclic electron flow influence on photosynthetic machinery resistance to stressors (Munekage et al., 2002, 2004; Zhang and Sharkey, 2009; Joliot and Johnson, 2011). This stimulation was observed under moderate actinic light (about 200 μmol·m^−2^·s^−1^) (Munekage et al., 2002, 2004). Miyake et al. (2004, 2005) showed that NPQ was strongly depended on cyclic electron flow when *EF(PSI)* / *EF(PSII)* > 1.2–1.3. Table 4 shows that VP activated *relative EF(C)* from 27 to 38% in pea (*EF(PSI)* / *EF(PSII)* increased from 1.37 to 1.61), i.e., activation of cyclic electron flow can influence NPQ. Figure 8 shows that *NPQ* and *relative EF(C)* were linearly connected that can be interpreted according to Miyake et al. (2004, 2005) as stimulation of NPQ by cyclic electron flow. Positive correlation between *NPQ* and *relative EF(C)* under control conditions also supports this hypothesis. Thus, it may be supposed that VP-induced cyclic electron flow activation stimulates NPQ and, thereby, increases of photosynthetic machinery resistance.

### Conflict of interest statement

The authors declare that the research was conducted in the absence of any commercial or financial relationships that could be construed as a potential conflict of interest.
